# Endoplasmic Reticulum Membrane Reorganization Is Regulated by Ionic Homeostasis

**DOI:** 10.1371/journal.pone.0056603

**Published:** 2013-02-15

**Authors:** Shankar Varadarajan, Kayoko Tanaka, Joshua L. Smalley, Edward T. W. Bampton, Maurizio Pellecchia, David Dinsdale, Gary B. Willars, Gerald M. Cohen

**Affiliations:** 1 MRC Toxicology Unit, University of Leicester, Leicester, United Kingdom; 2 Department of Biochemistry, University of Leicester, Leicester, United Kingdom; 3 Department of Cell Physiology and Pharmacology, University of Leicester, Leicester, United Kingdom; 4 Sanford-Burnham Institute, La Jolla, California, United States of America; Cornell University, United States of America

## Abstract

Recently we described a new, evolutionarily conserved cellular stress response characterized by a reversible reorganization of endoplasmic reticulum (ER) membranes that is distinct from canonical ER stress and the unfolded protein response (UPR). Apogossypol, a putative broad spectrum BCL-2 family antagonist, was the prototype compound used to induce this ER membrane reorganization. Following microarray analysis of cells treated with apogossypol, we used connectivity mapping to identify a wide range of structurally diverse chemicals from different pharmacological classes and established their ability to induce ER membrane reorganization. Such structural diversity suggests that the mechanisms initiating ER membrane reorganization are also diverse and a major objective of the present study was to identify potentially common features of these mechanisms. In order to explore this, we used hierarchical clustering of transcription profiles for a number of chemicals that induce membrane reorganization and discovered two distinct clusters. One cluster contained chemicals with known effects on Ca^2+^ homeostasis. Support for this was provided by the findings that ER membrane reorganization was induced by agents that either deplete ER Ca^2+^ (thapsigargin) or cause an alteration in cellular Ca^2+^ handling (calmodulin antagonists). Furthermore, overexpression of the ER luminal Ca^2+^ sensor, STIM1, also evoked ER membrane reorganization. Although perturbation of Ca^2+^ homeostasis was clearly one mechanism by which some agents induced ER membrane reorganization, influx of extracellular Na^+^ but not Ca^2+^ was required for ER membrane reorganization induced by apogossypol and the related BCL-2 family antagonist, TW37, in both human and yeast cells. Not only is this novel, non-canonical ER stress response evolutionary conserved but so also are aspects of the mechanism of formation of ER membrane aggregates. Thus perturbation of ionic homeostasis is important in the regulation of ER membrane reorganization.

## Introduction

Intracellular Ca^2+^ signaling is involved in the regulation of many cellular functions including those associated with growth, differentiation and apoptosis [1]. Sources of Ca^2+^ involved in regulating the cytoplasmic [Ca^2+^] ([Ca^2+^]_cyt_) include the extracellular fluid and the Ca^2+^ store in the endoplasmic reticulum (ER). This ER store is tightly controlled by a range of influx and efflux mechanisms, including the sarco/endoplasmic reticulum Ca^2+^ ATPase (SERCA), which is responsible for transferring Ca^2+^ into the ER lumen [2], [3]. Inhibitors of SERCA activity, such as thapsigargin (THG), prevent this transfer and deplete the store thereby elevating [Ca^2+^]_cyt_, as a consequence of a flux through leak channels [4]. The stimulated release of ER Ca^2+^ is brought about primarily by activation of the specific ER-resident channel proteins, the inositol 1,4,5-trisphosphate (IP_3_) receptors and ryanodine receptors [2], [3]. Depletion of ER Ca^2+^ stores triggers an influx of extracellular Ca^2+^ to provide a source for their replenishment. This store operated calcium entry (SOCE) is mediated by ER membrane proteins, such as STIM1 and STIM2, that detect reduced ER luminal [Ca^2+^] and interact with plasma membrane channel proteins, including ORAI and TRPC family members to mediate Ca^2+^ entry [5]–[8]. In addition to SOCE, other mechanisms of Ca^2+^ entry into the cell, including ARC (arachidonic acid regulated Ca^2+^ entry) have been identified [9].

Defects in intracellular Ca^2+^ homeostasis are a common occurrence in different stress conditions, where the functioning of the ER is disrupted. As a result, cells accumulate unfolded and misfolded proteins in the ER lumen. This causes ER stress, resulting in the activation of a coordinated intracellular signaling cascade called the unfolded protein response (UPR), in an effort to restore cellular homeostasis and integrity [10], [11]. The UPR causes a temporary arrest in global protein synthesis, while generating chaperones to deal with the unfolded proteins. However, when the extent of stress is overwhelming, the UPR signals the cell to undergo apoptosis by a number of mechanisms including up-regulation of proapoptotic BCL-2 family members and also by transferring Ca^2+^ to the mitochondria, which then orchestrates the intrinsic apoptotic pathway, eventually leading to the elimination of the stressed cell [12], [13].

Recently we described a novel cellular stress response characterized by a striking, but reversible, reorganization of ER membranes distinct from canonical ER stress and the UPR [14]. This ER membrane reorganization results in a dramatic redistribution and clustering of ER membrane proteins together with impaired ER transport and function. In our previous study, apogossypol, a putative broad spectrum BCL-2 family antagonist, was used as the prototype compound to induce ER membrane reorganization. Using connectivity mapping, we further established the widespread occurrence of this stress response identifying a wide range of structurally diverse chemicals from different pharmacological classes, including antihistamines, antimalarials, antiparasitics and antipsychotics that induce ER membrane reorganization [14]. Thus, ER membrane reorganization is a feature of a newly identified cellular stress pathway with potentially important consequences affecting the functioning of the ER.

In this study, we used hierarchical clustering to identify a group of ER membrane aggregating compounds that may act by perturbation of Ca^2+^ homeostasis. This was supported by the induction of ER membrane reorganization by calmodulin antagonists, SERCA inhibitors or over-expression of STIM1. However, induction of ER membrane reorganization by apogossypol required an influx of extracellular Na^+^, confirming the importance of ionic homeostasis in regulating ER membrane reorganization.

## Materials and Methods

### Cell culture

HeLa cells from ATCC (Middlesex, UK) were cultured in DMEM supplemented with 5 mM L-glutamine and 10% fetal calf serum (FCS) (all from Life Technologies, Paisley, UK). DT40 B cells, both IP_3_R (IP_3_ receptor) KO and IP_3_R1 reconstituted, from Prof. C. Taylor (University of Cambridge, UK) [15], were cultured in RPMI 1640 medium supplemented with 10% FCS and 1% heat inactivated chicken serum (Biosera Ltd., East Sussex, UK). *Schizosaccharomyces pombe* cells harbouring *rtn1-GFP* allele (KT4007 : *h^90^ ade6.M216 leu1.32rtn1-GFP-2×FLAG::Kan^R^*) were cultured either in rich YE medium or in minimal medium [16] supplemented with adenine and leucine.

### Reagents and plasmids

STIM1-YFP (Addgene plasmid 19754), ORAI1-YFP (Addgene plasmid 19756) and ORAI3-Myc (Addgene plasmid 16370) were from Dr. A. Rao (La Jolla Institute for Allergy and Immunology, La Jolla, CA, USA) [8], [17]. ORAI2-Myc was generated from a cDNA obtained by RT-PCR from total RNA of HeLa cells (using fwd: 5′-GAATTCATGAGTGC TGAGCTTAA-3′; rev: 5′-CTCGAGCAAGACCTGCAGGCTGCGCT-3′) and cloned into the *Eco*RI-*Xho*I sites of pcDNA3.1-Myc-His (Invitrogen, Carlsbad, CA, USA). BAP31 antibody was from Abcam (Cambridge, UK). ^45^Ca^2+^ (specific activity 32 mCi mg^−1^; 54 mCi mL^−1^) and scintillation fluid were from Perkin-Elmer Inc. (Cambridge, UK). The oil mixture used for collecting cells in the ^45^Ca^2+^ uptake and release experiments was from Dow Corning (Seneffe, Belgium). Apogossypol was synthesized as described [18]. TW37 was from Selleck Chemicals LLC (Houston, TX, USA). Calmidazolium, thapsigargin (THG), (N-(10-aminodecyl)-5-chloro-1-naphthalenesulfonamide hydrochloride) (A-7), 2-aminoethoxydiphenyl borate (2-APB) and LOE-908 were from Tocris Bioscience (Bristol, UK). Benzamil and all other reagents, unless mentioned otherwise, were from Sigma-Aldrich Co. (St. Louis, MO, USA).

### Transient overexpression, genomic tagging and siRNA knockdowns

For transient transfections, cells were transfected using TransIT-LT-1 transfection reagent (Mirus Bio LLC, WI, USA) and left for 48 h, according to the manufacturer's instructions. For siRNA knockdowns, cells were reverse-transfected with oligoduplexes (Life Technologies), using Interferin Reagent (Polyplus Transfection Inc, NY, USA), according to the manufacturer's protocol and processed 72 h after transfection. Cells were reverse-transfected with 10 nM of STIM1 (ID# S13563), ORAI1 (ID# S39560) or ORAI3 (ID# S41090). To visualize ER membrane aggregates in fission yeast, the *rtn1* gene (also known as *cwl1* [19]), which encodes a reticulon-like-domain-containing protein Rtn1 [20], was chromosomally tagged with GFP-2×FLAG as described previously [21] to generate the strain, KT4007 : *h^90^ ade6.M216 leu1.32 rtn1-GFP-2×FLAG::Kan^R^*.

### ^45^Ca^2+^ uptake and release experiments

^45^Ca^2+^ uptake and release experiments were carried out based on previously described methods [22]. Briefly, cells grown to confluence in one or more 175 cm^2^ flasks were washed and collected with a cell lifting buffer (0.9% (w/v) NaCl, 0.1% (w/v) EDTA and 10 mM HEPES, pH 7.4) and centrifuged at 1500 rpm for 3 minutes in a bench-top microfuge. The cells were washed twice with 3 mL of intracellular-like buffer (ICB), containing 120 mM KCl, 2 mM KH_2_PO_4_, 5 mM (NaH_2_COONa)_2._6H_2_O, 2.4 mM MgCl_2._6H_2_O, 20 mM HEPES, 5 mM ATP, pH 6.9 and enough EGTA to arrive at a [Ca^2+^] of 70–150 nM, as determined using fura-2 [23]. The cells were then permeabilized in 2 mL of ICB containing 0.1 mg mL^−1^ of saponin for 1 minute, followed by centrifugation at 1500 rpm for 2 minutes. For the ^45^Ca^2+^ uptake experiments, as an index of SERCA activity, the permeabilized cells were then resuspended in ICB with the appropriate concentrations of DMSO, apogossypol or THG, followed by the addition of 2.3 µCi mL^−1^ of ^45^Ca^2+^. The resuspended cells were then aliquoted and collected at the indicated time points by centrifugation at 14000 rpm for 1 minute. The cell pellets were rapidly resuspended in 0.5 mL of oil (1∶1 mixture of Dow Corning 556 Cosmetic Grade Fluid and Dow Corning 550 Fluid) and centrifuged for a further 1 minute at 14000 rpm. The sample tubes were then inverted to drain and air-dried at room temperature for 30 minutes. Finally, the pellets were dissolved in 1.1 mL of scintillant and associated ^45^Ca^2+^ determined by liquid scintillation counting. To determine Ca^2+^ release, cells were permeabilized as above with saponin and following collection, the intracellular stores were loaded by 20 minutes incubation with 2.3 µCi mL^−1^ of ^45^Ca^2+^. The release of ^45^Ca^2+^ was then determined by treatment of the cells with test reagents for the times indicated before collection of the cells and determination of the remaining associated ^45^Ca^2+^ exactly as described above.

### Ca^2+^ imaging

HeLa cells grown on coverslips were loaded with 2 µM fura-2 AM (Life Technologies) in extracellular medium (ECM), containing 121 mM NaCl, 5.4 mM KCl, 1.6 mM MgCl_2_, 1.8 mM CaCl_2_, 6 mM NaHCO_3_, 9 mM glucose, 25 mM HEPES, pH 7.4, in the dark at room temperature for 30 minutes. Cells were then washed and coverslips mounted on a Zeiss axiovert inverted microscope and maintained at 37°C in ECM. Epifluorescent images were collected with alternating excitation at 340 and 380 nm and emissions collected above 510 nm using a digital CCD camera (Orca ER, Hamamatsu, Hamamatsu City, Japan). Drugs were added at indicated time points. For measurement of SOCE, cells were incubated in nominally Ca^2+^ free ECM and treated as indicated, followed by addition of CaCl_2_ to the ECM to a final concentration of 1.8 mM to allow SOCE. For all experiments, changes in [Ca^2+^]_cyt_ were measured in 20 individual cells in each of at least three separate experiments, unless otherwise specified.

### Elimination media experiments in yeast

To investigate the role of cationic influx in ER membrane reorganization, *S. pombe* cells, harbouring *rtn1-GFP* at its own chromosomal locus were exposed to the different chemicals with modifications of the minimal media [16]. Briefly, yeast cells cultured in rich yeast extract medium [16] to a density of 1×10^7^ cells mL^−1^ at 30°C were extensively washed and diluted with either low Ca^2+^ medium (minimal media minus the CaCl_2_ and supplemented with 20 mM EGTA), low K^+^ medium (minimal media minus KCl and potassium hydrogen phthalate), low Mg^2+^ medium (minimal media minus the MgCl_2_) or low Na^+^ medium (minimal media minus Na_2_HPO_4_ and Na_2_SO_4_) to a density of 2×10^6^ cells mL^−1^. They were then exposed to drugs for 2 h, followed by image acquisition.

### Microarray analysis and hierarchical clustering

We previously identified a subset of chemicals that induce ER membrane reorganization [14]. To tease apart potential functional differences between chemicals that induce ER membrane reorganization, we performed hierarchical clustering analysis using gene-expression data for ER membrane aggregating chemicals following a 6-hour exposure in MCF-7 cells. We utilized both in-house generated microarray data and Connectivity Map datasets for this application. For generating the in-house microarray data, total RNA extracted from MCF-7 cells exposed to different agents was used to make biotin-labeled cRNA using the Illumina TotalPrep RNA amplification kit, hybridized to an Illumina HumanHT-12 BeadChip array, Cy3 labeled and scanned using an Illumina BeadArray Reader (all from Illumina, Hayward, CA, USA). Microarray data normalization and analyses were carried out using ArrayTrack software (NCTR/FDA, Jefferson, AR, USA), and the data sets were compared by Welch's t-test. Microarray data were normalized using a quantile normalization method and filtered to remove genes with low mean channel intensities (<250). The top 50 most differentially expressed genes across all datasets were used for hierarchical clustering, using a Pearson correlation metric and average link clustering.

### Microscopy

For immunofluorescent staining, cells grown on coverslips were fixed with 4% (w/v) paraformaldehyde, permeabilized with 0.5% (v/v) Triton X-100 in PBS and followed by incubations with primary antibodies, the appropriate fluorophore-conjugated secondary antibodies, mounted on glass slides and subjected to confocal microscopy on a Zeiss LSM510 (Cambridge, UK). Imaging of the yeast cells was conducted at 30°C using a Leica SP5 laser scanning confocal microscope. Cells were immobilized on glass bottom dishes (MatTek Corporation, Ashland, MA, USA) coated with lectin from *Bandeiraea simplicifolia* (Sigma-Aldrich Co.) and incubated with appropriate media. Images along the Z axis were taken every 0.5–0.6 µm to fully cover the thickness of the cell. Obtained images were processed by Huygens Essential (Scientific Volume Imaging, Hilversum, Holland), a deconvolution software, and maximum Z-projection images were generated by Image J (NIH, Bethesda, MD, USA). For electron microscopy, cells were fixed and processed as previously described [14]. Electron micrographs were recorded using an ES1000W CCD camera and Digital Micrograph software (Gatan, Abingdon, UK) with a Zeiss 902A electron microscope or with a Megaview 3 digital camera and iTEM software (Olympus Soft Imaging Solutions GmbH, Münster, Germany) in a Jeol 100-CXII electron microscope (Jeol UK Ltd., Welwyn Garden City, UK).

## Results

### Hierarchical clustering reveals a possible role for altered Ca^2+^ homeostasis in ER membrane reorganization

**Figure 1:**
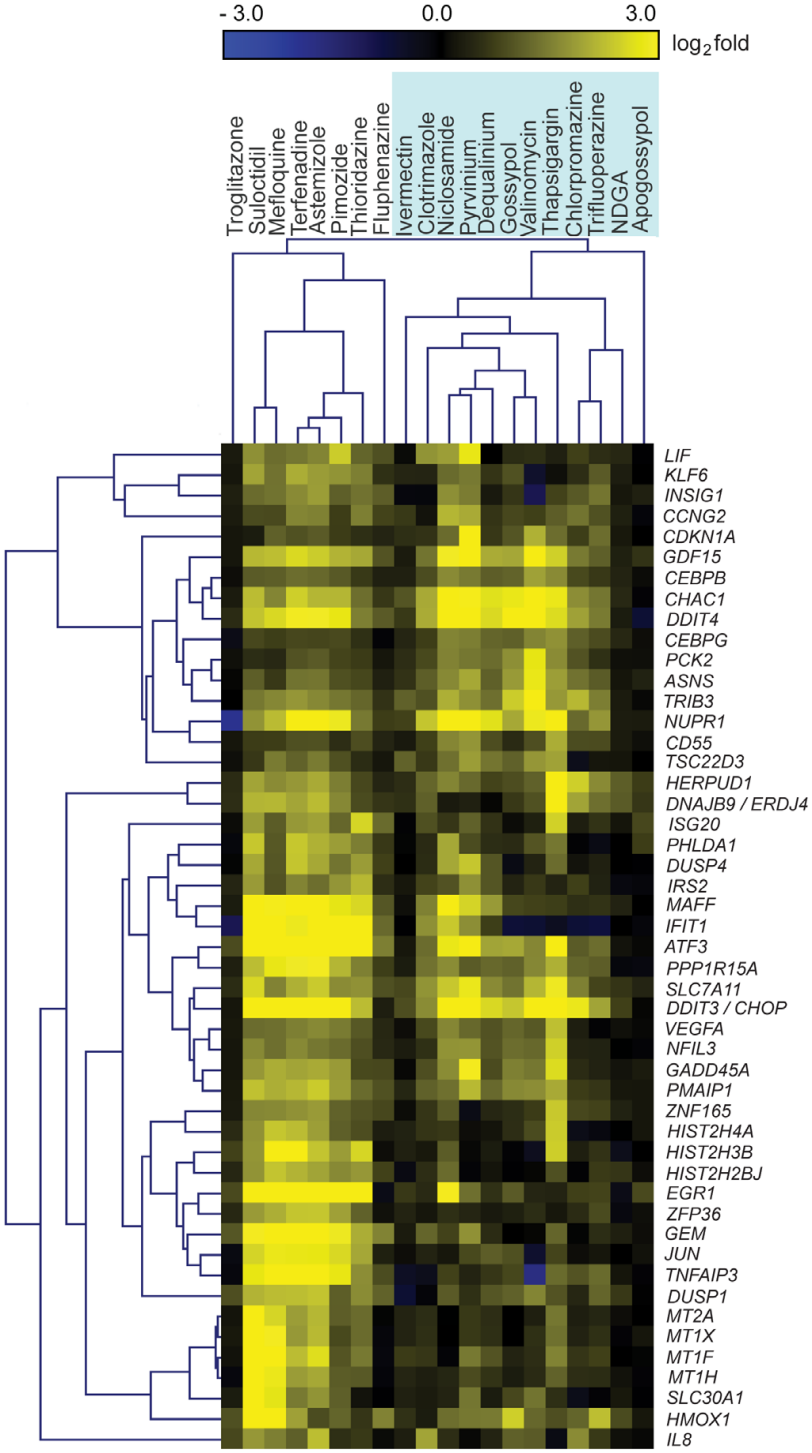
Hierarchical clustering reveals a possible role for Ca^2+^ homeostasis in ER membrane reorganization. Heat map analysis comparing the top 50 differentially expressed genes following the indicated drug treatments as available in the connectivity map reference database. Yellow and blue indicate up or down regulation, respectively. Hierarchical clustering was used to analyze microarray data for chemicals known to induce ER membrane reorganization. The microarray data were normalized, compared with the equivalent control samples, and filtered by mean channel intensity (<250) and mean log_2_ fold changes across all the data sets (<1.8). Trends in the resulting gene expression matrix were analyzed, using a Pearson correlation metric and average link clustering. Based on transcriptional trends, two distinct groups of chemicals were identified. Apogossypol clustered with a number of chemicals that are known to disrupt Ca^2+^ homeostasis (shaded in blue box).

Using microarray analysis and connectivity mapping, we previously identified a diverse range of chemicals that induce ER membrane reorganization thereby demonstrating the widespread occurrence of this novel cellular response [14]. To identify a possible common mechanism shared by these chemicals in the induction of ER membrane aggregates, we further analyzed the connectivity map microarray datasets for these chemicals [24]. Using hierarchical clustering, we identified two distinct chemical groups, potentially indicating functional chemical differences (Figure 1). Apogossypol clustered with several chemicals, including THG, ivermectin and chlorpromazine, all of which disrupt Ca^2+^ homeostasis, suggesting a possible role for this in ER membrane reorganization (Figure 1 and Table 1). Moreover, we have previously observed that preventing the reuptake of released Ca^2+^ by exposing cells to several SERCA inhibitors, including THG, 2,5-di-*t*-butyl-1,4-benzohydroquinone (BHQ) and cyclopiazonic acid (CPA) resulted in ER membrane reorganization [14]. In this regard, several ER membrane reorganizing compounds previously identified by connectivity mapping, including several antipsychotic phenothiazines, are also known to inhibit SERCA (Figure 1 and Table 1), thus potentially linking ER membrane reorganization to the inhibition of SERCA activity and altered Ca^2+^ handling.

**Table 1:** Perturbation of Ca^2+^ homeostasis by compounds clustered with apogossypol.

Compounds clustered with apogossypol	Activity	Extent of ER membrane reorganization	Effects on Ca^2+^ homeostasis	References
Ivermectin	Antiparasitic	+++	SERCA inhibitor	[47]
Clotrimazole	Antimyotic	+++	SERCA inhibitor	[48]
Niclosamide	Antihelminthic	+	Not known	
Pyrvinium	Antihelminthic	+	Effects Ca^2+^ channels	[49]
Dequalinium	Antimalarial/Antiseptic	+	Inhibits intracellular Ca^2+^ uptake	[50]
Gossypol	Antifertility/Antimalarial	+++	Ca^2+^ release from rat liver mitochondria	[51]
Valinomycin	Antibiotic	+	Inhibits intracellular Ca^2+^ uptake	[52]
Thapsigargin	Sesquiterpene lactone	+++	SERCA inhibitor	[4]
Chlorpromazine	Antipsychotic phenothiazine	+	Inhibits SOCE	[53]
Trifluoperazine	Antipsychotic phenothiazine	++	Calmodulin and SERCA inhibitor	[54], [55]
NDGA	Antioxidant	+++	SERCA inhibitor	[56]

Listing of the eleven compounds that clustered with apogossypol in Figure 1 and their pharmacological activities. HeLa cells after 4 h exposure to ivermectin (20 µM), clotrimazole (50 µM), niclosamide (50 µM), pyrvinium (20 µM), dequalinium (10 µM), gossypol (10 µM), valinomycin (20 µM), thapsigargin (10 µM), chlorpromazine (20 µM), trifluoperazine (20 µM) or NDGA (50 µM) exhibited varying levels of ER membrane reorganization, from minimal (**+**) to extensive reorganization (**+++**). Ten of the eleven compounds have been described to perturb cellular Ca^2+^ homeostasis by different mechanisms.

### Apogossypol-mediated release of intracellular Ca^2+^ is not directly linked to ER membrane reorganization

**Figure 2:**
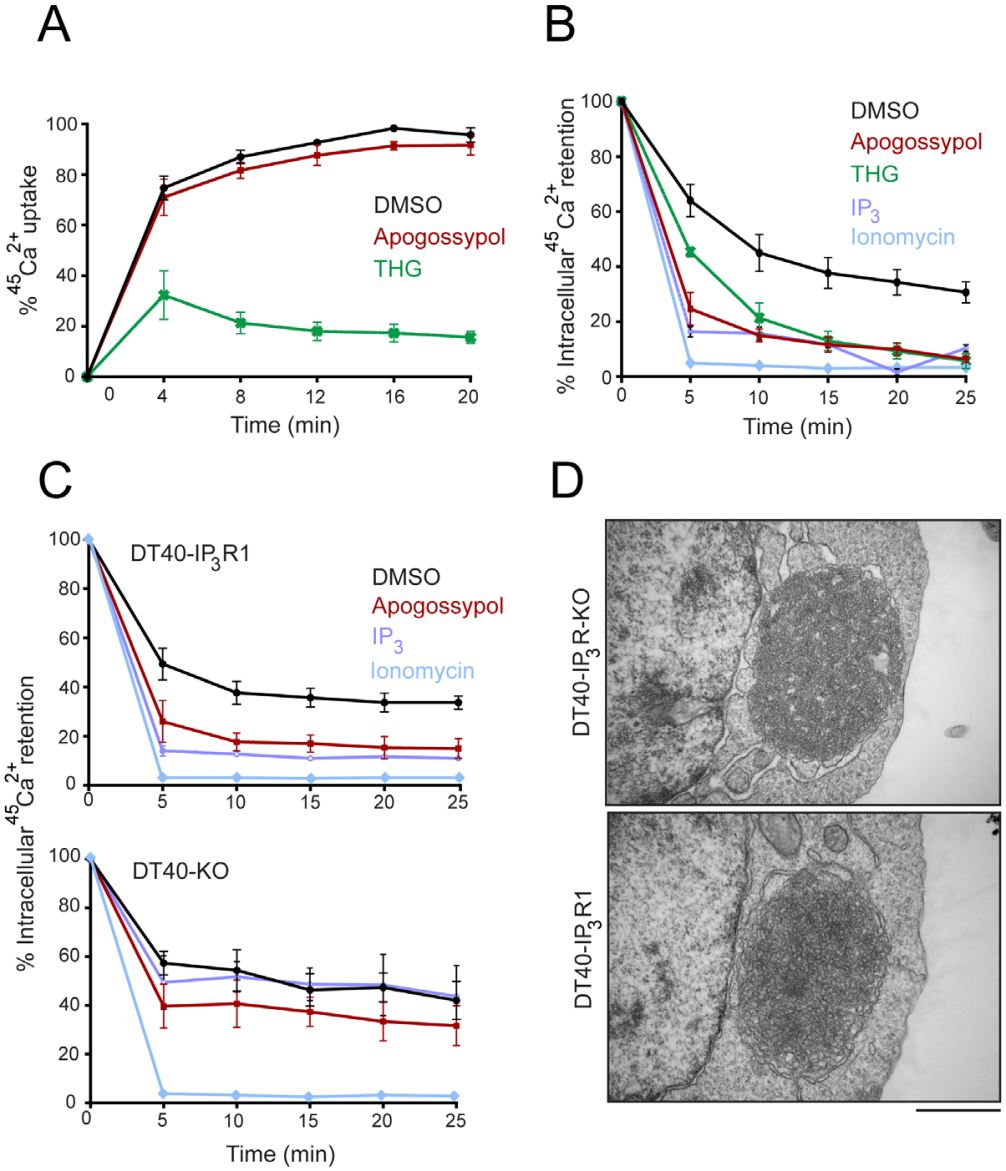
Apogossypol-induced ER membrane reorganization is not due to depletion of intracellular Ca^2+^ stores. (A) Permeabilized HeLa cells, exposed to DMSO (control), apogossypol (10 µM), or THG (10 µM), were labelled with ^45^Ca^2+^ and samples collected at the indicated times to assess ^45^Ca^2+^ uptake. (B) Permeabilized HeLa cells labelled with ^45^Ca^2+^ for 20 minutes, were exposed to DMSO (control), apogossypol (10 µM), THG (10 µM), IP_3_ (3 µM) or ionomycin (1 µM) for the indicated times and samples collected and assessed for the extent of ^45^Ca^2+^ release. (C) Experiments were carried out as in (B) except using DT40 cells, that either lack all 3 isoforms of IP_3_ receptors (DT40 KO) or were reconstituted with IP_3_R1 (DT40-IP_3_R1). (D) Extensive ER membrane reorganization, as assessed by electron microscopy (scale bar, 1 µm) was observed in DT40- IP_3_R1 and DT40 KO cells, exposed to apogossypol (10 µM) for 4 h. Error bars shown in A–C represent the standard error of the mean.

To assess if inhibition of SERCA activity was critical for ER membrane reorganization, we monitored the extent of uptake of radiolabeled Ca^2+^ (^45^Ca^2+^) into the intracellular stores of HeLa cells, following exposure to different agents. THG blocked Ca^2+^ uptake, in agreement with its known activity as an irreversible inhibitor of SERCA [4]. However, apogossypol did not block Ca^2+^ uptake, suggesting that apogossypol induced ER membrane reorganization independent of reduced SERCA activity (Figure 2A). Nevertheless, both apogossypol and THG caused a marked stimulation of ^45^Ca^2+^ efflux from intracellular stores similar to that induced by IP_3_, while ionomycin (used as a positive control) caused a complete release (Figure 2B). Since the extent of apogossypol-mediated ^45^Ca^2+^ efflux was very similar to that mediated by IP_3_, we speculated that apogossypol-mediated Ca^2+^ release may also occur *via* the IP_3_ receptors. To explore this possibility, we used either chicken DT40 B lymphocytes that lack all three isoforms of the IP_3_ receptor (DT40-KO) or cells that were reconstituted with IP_3_ receptor isoform 1 (DT40-IP_3_R1) [15]. Both apogossypol and IP_3_ induced extensive release of intracellular Ca^2+^ (∼85%) in permeabilzed DT-40-IP_3_R1 cells (Figure 2C, top panel), whereas in the absence of IP_3_ receptors the IP_3_-mediated Ca^2+^ efflux was essentially reduced to control levels, and the release mediated by apogossypol was somewhat reduced (Figure 2C, bottom panel). Moreover, neither caffeine nor ryanodine caused any efflux of intracellular Ca^2+^, thus excluding any involvement of ryanodine receptors (data not shown). These results suggest that apogossypol-stimulated Ca^2+^ efflux from the ER is partially mediated by IP_3_ receptors. Nevertheless, apogossypol induced a similar degree of ER membrane reorganization in both DT40KO and IP_3_R1 cells (Figure 2D), suggesting that the release of ER Ca^2+^ *via* IP_3_Rs is not responsible for apogossypol-induced ER membrane reorganization.

### ER membrane reorganization is associated with an altered handling of Ca^2+^

**Figure 3:**
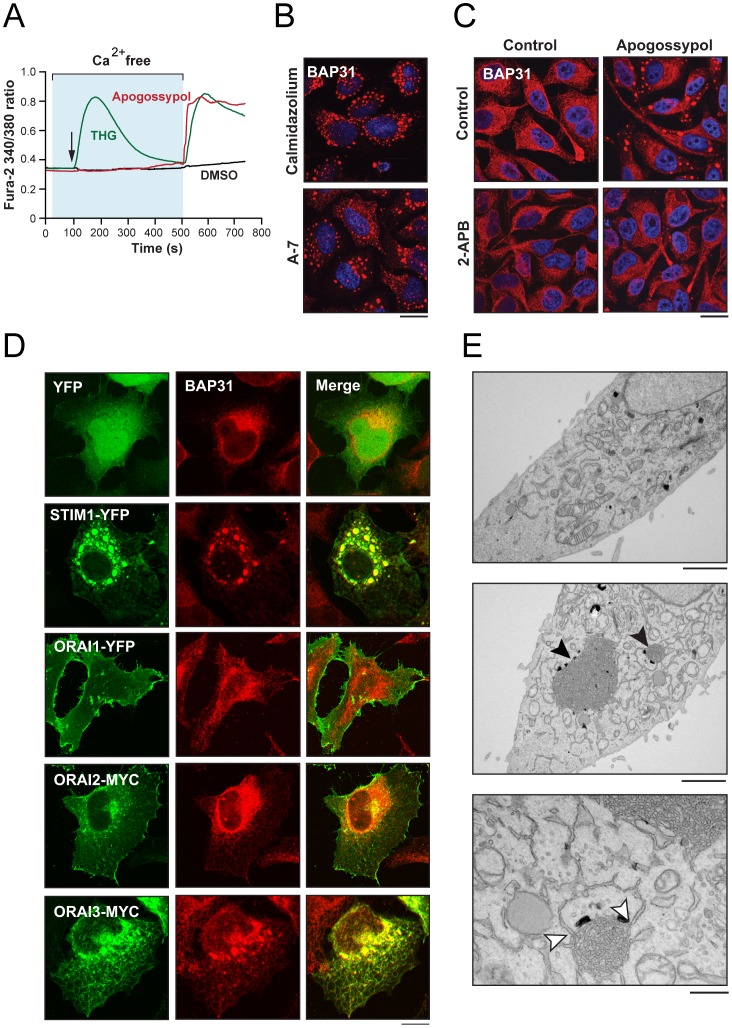
ER membrane reorganization may be due to enhanced Ca^2+^ entry. (A) Fura-2-loaded HeLa cells were exposed to either apogossypol (10 µM) or THG (10 µM) in Ca^2+^-free media. This was followed by addition of extracellular Ca^2+^. Changes in fura-2 fluorescence were measured throughout as an index of changes in [Ca^2+^]_cyt_. The graph represents the mean from 3 independent experiments with 20 cells recorded in each experiment. (B) Extensive ER membrane reorganization, assessed by BAP31 staining, was observed in HeLa cells exposed for 4 h to the calmodulin inhibitors, calmidazolium (10 µM) and A-7 (20 µM) (scale bar, 20 µm). (C) Apogossypol-mediated ER membrane reorganization was abolished when HeLa cells were pretreated for 1 h with 2-APB (10 µM) (scale bar, 20 µm). (D) Transient overexpression of YFP, STIM1-YFP, ORAI1-YFP, ORAI2-Myc or ORAI3-Myc and immunostaining with BAP31 antibody, revealed that STIM1-YFP caused extensive ER membrane reorganization, similar to that observed following apogossypol (scale bar, 10 µm). (E) Ultrastructure of STIM1-YFP expressing HeLa cells showing STIM1-mediated reorganization of ER membranes (lower two panels). The bottom panel (scale bar, 5 µm) shows a detail of one of two regions of pronounced ER membrane organizations (indicated by black arrows in the middle panel). It also shows a clear continuity of membranes with the rest of the ER (white arrows). The upper panel shows a comparable area of control transfected HeLa cell (the scale bar in the top 2 panels is 2 µm).

When cells were treated with THG in the absence of extracellular Ca^2+^, re-addition of Ca^2+^ to the outside of the cells resulted in a marked increase in [Ca^2+^]_cyt_ (Figure 3A), consistent with store depletion and stimulation of SOCE. Apogossypol also evoked a similar increase in [Ca^2+^]_cyt_ under these experimental conditions (Figure 3A). However, this occurred in the absence of an initial apogossypol-mediated increase in [Ca^2+^]_cyt_ implying Ca^2+^ influx *via* either a non-SOCE pathway or alternatively SOCE following store depletion in the absence of a detectable increase in [Ca^2+^]_cyt_. Nevertheless, ER membrane reorganization mediated by both apogossypol and THG was associated with Ca^2+^ influx. We therefore investigated if enhanced Ca^2+^ influx mediated by other agents would result in ER membrane reorganization. Several calmodulin antagonists, including calmidazolium, A-7 and trifluoperazine, reported to induce Ca^2+^ influx or alter Ca^2+^ handling [25], [26], also resulted in extensive ER membrane reorganization (Figure 3B) [14]. Moreover, 2-APB, an inhibitor of SOCE and potentially IP_3_ receptors, abolished apogossypol induced ER membrane reorganization (Figure 3C) [27], [28]. To further understand if ER membrane reorganization could result from enhanced SOCE, we overexpressed STIM1 and ORAI 1–3 proteins, which are critical for the induction of SOCE [6], [7]. Overexpression of STIM1-YFP resulted in excessive clustering of ER membranes in >60% of the transfected cells, which co-localized with the BAP31-positive ER membrane aggregates (Figure 3D). In contrast, overexpressed ORAI 1 and 2 localized to the plasma membrane and did not result in ER membrane reorganization (Figure 3D). ORAI3 localized to and caused minor clustering of ER membranes (Figure 3D). The excessive clustering of STIM1-YFP positive ER aggregates was most likely a result of overexpression, as cells expressing very low levels of STIM1-YFP failed to produce similar aggregates and maintained normal ER ultrastructure (data not shown), in agreement with previous reports [7], [29], [30]. However we cannot completely exclude the possibility that expression of YFP may in some way facilitate aggregation of STIM1. Furthermore, ER membrane reorganization was not observed following overexpression of other ER resident proteins, including SEC22-CFP, KDEL-RFP and SEC61-GFP ([14] and data not shown), implying that the marked ER ultrastructural change following STIM1 transfection was probably not an overexpression artifact but was more likely due to a functional activity of the overexpressed STIM1 protein, suggesting a potential involvement of STIM1 and/or SOCE in ER membrane reorganization.

To further exclude the possibility that ER membrane reorganization following STIM1-YFP overexpression was a consequence of the nonspecific aggregation of the YFP-fused protein, resulting in OSER (organized smooth ER) [31]–[33], we examined the ultrastructure of these cells. The STIM1-YFP positive ER aggregates clearly resembled the patches of disorganized ER membranes induced by apogossypol (Figure 3E) and were clearly distinct from OSER induced by over-expression of ER resident proteins, including the IP_3_ receptor and hydroxyl-methylglutaryl (HMG)-CoA reductase [31]–[33]. We therefore wished to test the possibility raised by these data that apogossypol-induced ER membrane reorganization may be due to an enhanced store mediated influx of Ca^2+^, although we cannot exclude the involvement of a non-SOCE Ca^2+^ entry.

### SOCE is not essential for ER membrane reorganization

**Figure 4:**
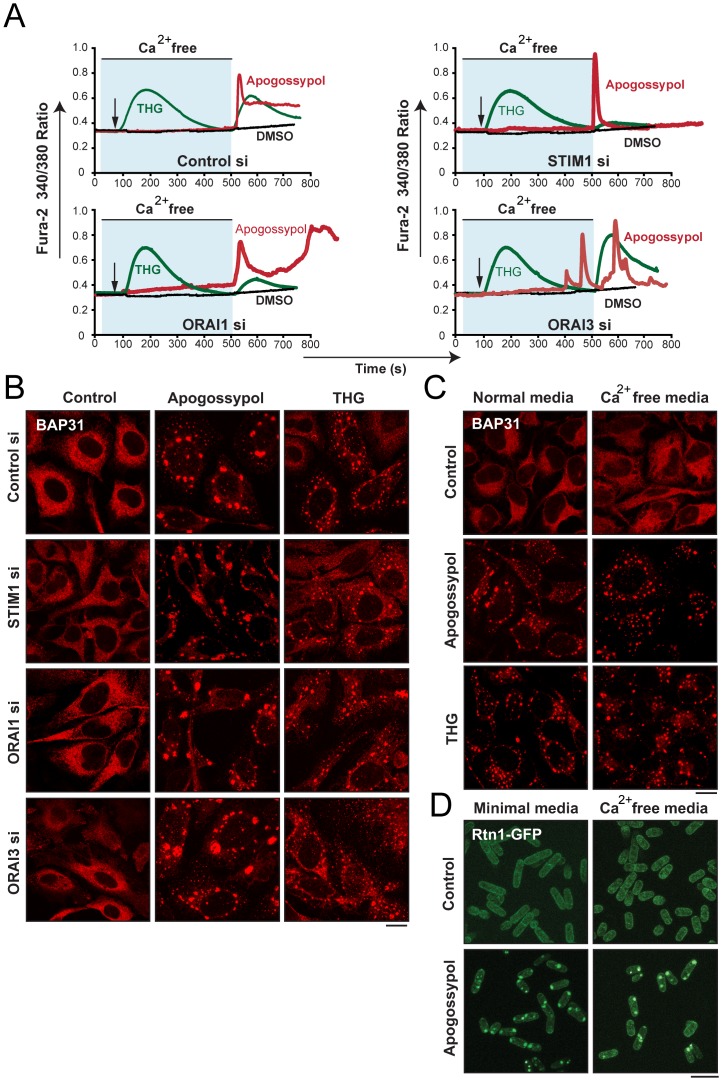
Enhanced Ca^2+^ influx is not critical for apogossypol-mediated ER membrane reorganization. (A) Down-regulation of STIM1, ORAI1 or ORAI3, using RNA interference for 72 h, had no significant inhibitory effects on apogossypol-induced Ca^2+^ entry, as measured by the maximal ratio change in fura-2 fluorescence in Ca^2+^-free conditions followed by addition of extracellular Ca^2+^. In contrast, THG-mediated SOCE was dramatically reduced following STIM1 and ORAI1 siRNA but not with ORAI3 siRNA. The graph for THG represents the mean from 3 independent experiments with 20 cells recorded in each experiment. Following apogossypol, the effects were not uniform (the cells behaving inconsistently, often exhibiting multiple peaks) and the graph represents data from a single cell in one of three independent experiments. (B) HeLa cells, transfected for 72 h with siRNA against STIM1, ORAI1 or ORAI3, were exposed to apogossypol (10 µM) for 4 h and immunostained with BAP31 antibody to assess ER membrane reorganization (scale bar, 20 µm). (C) HeLa cells, exposed to apogossypol (1 µM) or THG (5 µM) for 1 h, in serum-free normal DMEM or in serum- and Ca^2+^-free DMEM, were immunostained with BAP31 antibody to assess ER membrane reorganization (scale bar, 20 µm). (D) *S. pombe*, harboring *rtn1-GFP* at its endogenous *rtn1* locus to visualize the ER membranes, were grown for 2 h in minimal media or Ca^2+^-free media (supplemented with 20 mM EDTA), in the presence or absence of apogossypol (10 µM) (scale bar, 10 µm).

To ascertain the potential role of this Ca^2+^ influx, we silenced the genes required for SOCE and assessed apogossypol- and THG-induced Ca^2+^ influx and ER membrane reorganization. Down-regulation of STIM1 and ORAI1 almost totally abolished THG-mediated SOCE but had little inhibitory effect on apogossypol-mediated Ca^2+^ influx (Figure 4A). However, it should be noted that following STIM1 or ORAI1 knockdown, cells exposed to apogossypol behaved inconsistently, exhibiting a variety of patterns of single and multiple peaks, making it difficult to assess the extent of Ca^2+^ influx, although this was clearly not blocked. Down regulation of ORAI3 did not block THG-mediated SOCE and again resulted in multiple and variable spikes in [Ca^2+^]_cyt_ following exposure to apogossypol (Figure 4A, bottom right panel). Despite these changes in the extent and pattern of THG- or apogossypol-induced [Ca^2+^]_cyt_ elevations, silencing either STIM1, ORAI1 or ORAI3 genes had no inhibitory effect on apogossypol- or THG-mediated ER membrane reorganization (Figure 4B), which implied that these proteins are not involved in either apogossypol- or THG-induced ER membrane reorganization. Importantly, ER membrane reorganization was apparent even in cells exposed to apogossypol or THG in Ca^2+^-free conditions, further supporting the suggestion that an influx of extracellular Ca^2+^ is not critical for the ER changes (Figure 4C). To confirm these observations, we exploited the evolutionary conserved nature of this phenomenon, and used fission yeast. Similar to the effects observed in mammalian cells, apogossypol induced ER membrane reorganization in fission yeast both in minimal media and Ca^2+^-free media (Figure 4D), confirming that an enhanced Ca^2+^ influx is not important for the induction of ER membrane reorganization in either human or yeast cells.

### Influx of Na^+^ is critical for apogossypol-induced ER membrane reorganization

**Figure 5:**
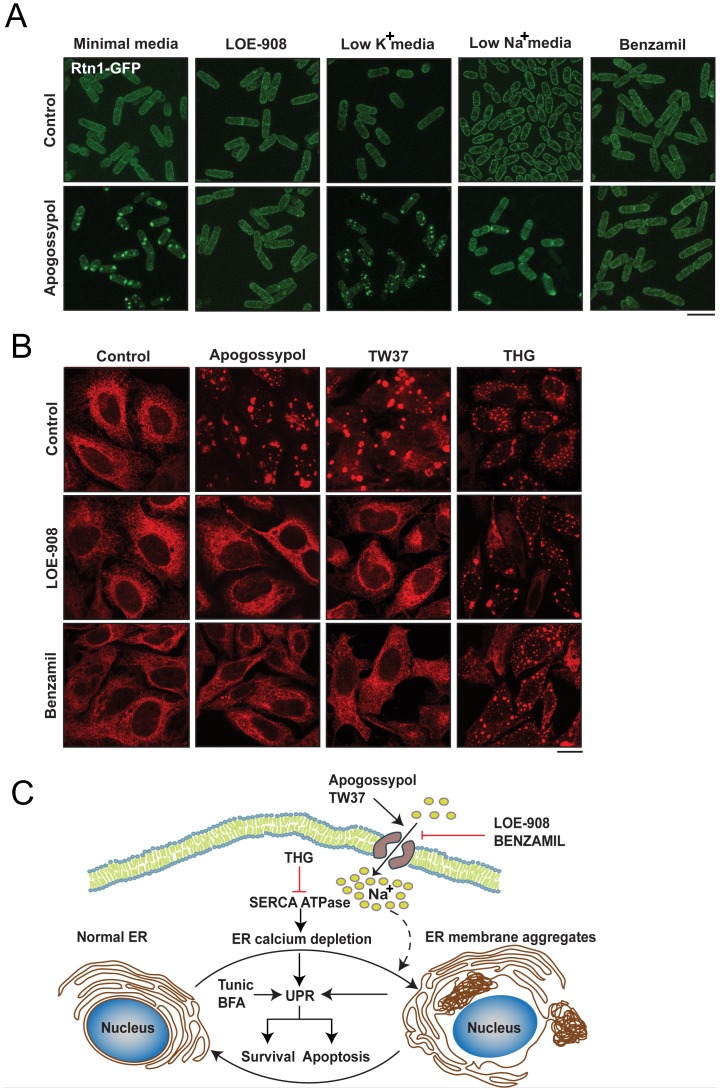
Apogossypol induces ER membrane reorganization *via* an enhanced influx of Na^+^. (A) *S. pombe* cells, harbouring *rtn1-GFP*, exhibited extensive ER membrane reorganization following exposure for 2 h to apogossypol (10 µM), when grown in minimal media, or minimal media lacking K^+^. Apogossypol-mediated ER membrane reorganization was abolished when cells were suspended in Na^+^-free media or exposed to either LOE-908 (10 µM) or benzamil (100 µM) 30 minutes before exposure to apogossypol (scale bar, 10 µm). (B) Influx of Na^+^ is required for ER membrane reorganization induced by some but not all agents in HeLa cells. Thus, LOE-908 (10 µM) or benzamil (100 µM) abolished apogossypol- (10 µM) and TW37- (50 µM)-induced membrane aggregates but not those induced by THG (10 µM) (scale bar, 20 µm). (C) Scheme summarizing the formation, regulation and consequences of ER membrane reorganization. Exposure to stress conditions, such as inhibition of the BCL-2 family (apogossypol, TW37) or SERCA (THG) induced ER membrane reorganization (right hand panel). Multiple mechanisms, including an influx of Na^+^, through LOE-908- and benzamil-sensitive Na^+^ channels, and ER Ca^2+^ depletion or altered Ca^2+^ handling regulate ER membrane reorganization. These membrane aggregates are reversible and occur independent of canonical ER stress, as tunicamycin (Tunic) and brefeldin A (BFA), two inducers of canonical ER stress, do not result in ER membrane reorganization [14]. However, at later times, the extensive ER membrane reorganization may eventually lead to canonical ER stress and the UPR, thus placing ER membrane reorganization upstream and/or independent of the UPR.

Although apogossypol induced extensive ER membrane reorganization in Ca^2+^-free conditions, LOE-908, a broad spectrum cation channel inhibitor [34], completely blocked apogossypol-induced ER membrane reorganization in fission yeast (Figure 5A), which suggested a requirement of a cation influx other than Ca^2+^. A series of elimination experiments revealed that removal of extracellular Na^+^, but not K^+^, Mg^2+^ or Ca^2+^, markedly decreased apogossypol-induced ER membrane reorganization (Figure 5A and data not shown). This was further supported by the observation that benzamil, a potent Na^+^-channel blocker [35], also abolished the ability of apogossypol to induce ER membrane aggregates (Figure 5A). LOE-908 and benzamil also inhibited apogossypol-induced ER membrane reorganization in HeLa cells (Figure 5B), suggesting an evolutionary conserved requirement for Na^+^ influx in the induction of ER membrane aggregates. Furthermore, both these compounds inhibited TW37- but not THG-induced ER membrane reorganization (Figure 5B). Like apogossypol, TW37 is also a broad spectrum BCL-2 family antagonist [36], [37]. Taken together these results demonstrate that ER membrane reorganization induced by broad spectrum BCL-2 family inhibitors is primarily initiated by perturbation of Na^+^ homeostasis.

## Discussion

In our previous study we characterized a novel cellular stress response, involving a rapid reorganization of ER membranes that is distinct from the canonical ER stress response and the UPR [14]. Using microarray analysis and connectivity mapping, we also demonstrated that this is a common cellular response to a wide range of structurally diverse chemicals from different pharmacological classes [14]. Here, further investigation revealed that many of these agents may perturb Ca^2+^ homeostasis (Figure 1 and Table 1). Moreover, ER network formation is accompanied by changes in Ca^2+^ homeostasis [38], [39], which occurs due to either enhanced influx of extracellular Ca^2+^, inhibition of the reuptake of cytosolic Ca^2+^ into ER stores or pronounced release from intracellular Ca^2+^ stores. In the experiments showing that three structurally distinct SERCA inhibitors induced ER membrane reorganization [14], a concentration of THG was used (5–10 µM) that was higher than that generally used to inhibit SERCA (e.g., 2 µM), which raised the possibility that other properties of these molecules in addition to SERCA inhibition may be required for them to induce these ultrastructural changes. Nevertheless, experimental support for a role for Ca^2+^ in ER membrane reorganization was provided by our findings that exposure to inhibitors of calmodulin or over-expression of STIM 1, an ER resident Ca^2+^ sensor essential for SOCE, induced ER membrane reorganization (Figure 3). Furthermore, 2-APB, an inhibitor of SOCE [27], [28], blocked apogossypol-induced ER membrane reorganization (Figure 3), thereby implicating SOCE as a mediator of the ER changes. However, THG caused a profound induction of ER membrane reorganization even when SOCE was inhibited by down-regulation of either STIM1 or ORAI1 (Figure 4). In addition, removal of extracellular Ca^2+^ had no impact on ER membrane reorganization in response to any of the inducers (Figure 4C and data not shown), suggesting that Ca^2+^ influx across the plasma membrane is not required. It is possible, therefore, that effects of 2-APB on aspects other than SOCE [27] may underlie its inhibitory action on apogossypol-induced changes. Further, as 2-APB did not inhibit ER membrane reorganization in response to other agents, including THG, this suggests that such structural changes may be mediated by multiple mechanisms or alternatively, 2-APB is unable to block later steps in the pathway that are triggered by these agents.

Previously we had shown that this novel form of non-canonical ER stress was evolutionary conserved as ER membrane reorganization was observed in human, mouse and Chinese hamster cells as well as in the fission yeast, *S. pombe* [14]. We have extended this aspect of our previous study to show that ER membrane reorganization also occurs in chicken cells (Figure 2D). More importantly, not only is the phenomenon of ER membrane reorganization evolutionary conserved, but some aspects of the mechanism of the formation of ER membrane aggregates are also conserved between man and fission yeast. This was most clearly illustrated by our finding that apogossypol-induced ER membrane reorganization required an influx of extracellular Na^+^ both in a human tumor cell line (HeLa) and in fission yeast (Figure 5). A similar requirement for extracellular Na^+^ influx was also observed with TW37, a related broad spectrum BCL-2 family antagonist, but not for THG. Taken together these results demonstrate that ER membrane reorganization induced by broad spectrum BCL-2 family inhibitors is primarily regulated by perturbation of Na^+^ homeostasis, although other mechanisms including altered ER Ca^2+^ handling may also be important and may even be a common thread underlying ER membrane reorganization. In this respect it is interesting to note that the BCL-2 family proteins have been proposed to regulate ER Ca^2+^, although there is little agreement on the precise mechanism [40]–[43]. It is possible that there may be some relationship between the BCl-2 family, Ca^2+^and Na^+^ homeostasis.

Inhibitors of apogossypol-induced ER membrane reorganization, including 2-APB and LOE 908, could be exerting their activity by blocking the Na^+^ influx *via* TRP and other Na^+^ channels [27], [44], [45], which could explain why these inhibitors failed to abolish the effects of THG, which may be mediated directly at the level of the ER. This would also suggest that apogossypol enhances extracellular Na^+^ influx and we therefore attempted to characterize the mechanism of such influx in fission yeast. This was seen as a more tractable model than mammalian cells, as Na^+^ influx in mammalian cells is complex and is carried out by several mechanisms, including Na^+^/Ca^2+^ exchangers, Na^+^/H^+^ exchangers and several other symporters [46]. In *S. pombe*, only three such genes, *sod2* (SPAC977.10), SPAC15A10.06 and SPAC3A12.06c, can be identified in PomBase, a comprehensive database for fission yeast (www.pombase.org). However, single deletion mutants of these genes had no apparent inhibitory effects on apogossypol-mediated ER membrane reorganization (Figure S1), thereby preventing a conclusive demonstration of the most important channels. Moreover, THG failed to induce ER membrane reorganization in fission yeast (data not shown), most likely due to the lack of SERCA in *S. pombe*.

In summary we have extended our previous study on this novel form of ER stress and demonstrated that some features of the mechanism of formation of ER membrane reorganization are also evolutionary conserved between man and yeast. We now show that an altered cellular ion homeostasis is associated with the ability of a range of chemicals to induce ER membrane reorganization. Given the structural diversity of these chemicals, it is unlikely that they precipitate ER membrane reorganization through identical mechanisms. Indeed here we show that the broad-spectrum BCL-2 family inhibitors, apogossypol and TW37, mediate ER membrane reorganization by a mechanism dependent on the influx of extracellular Na^2+^ through benzamil- and LOE-908-sensitive channels. In contrast, inhibition of SERCA by THG and altered cellular Ca^2+^ homeostasis by the calmodulin inhibitors A-7 and calmidazolium resulted in ER membrane reorganization that at least for THG was independent of extracellular Na^+^ influx. Although these data suggest that different mechanisms precipitate the events responsible for ER membrane reorganization, they do not rule out a common final pathway. It is conceivable, for example, that altered concentrations of Na^+^ in the sub-plasmalemmal region or immediately adjacent to the ER are able to perturb cellular Ca^2+^ handling, particularly by the ER, and it is this that drives the ER membrane reorganization.

## Supporting Information

Figure S1 **Deletion of sodium channels in fission yeast did not inhibit apogossypol-mediated ER membrane reorganization.** Fission yeast strain KT4007 (*h^90^ ade6.M216 leu1 rtn1-GFP-2×FLAG::Kan^R^*) carrying GFP-2×FLAG tagged *rtn1* gene and subjected to chromosomal gene deletion of either SPAC977.10 (*sod2*), SPAC15A10.06 or SPAC3A12.06c, still exhibited extensive ER membrane reorganization with apogossypol. (scale bar, 10 µm). Some sort of change in cell morphology (overall larger cells) was noticeable upon deletion of SPAC15A10.06.
(TIFF)
