# Supplementary figures and images for: Endoplasmic Reticulum Membrane Reorganization Is Regulated by Ionic Homeostasis
Source: PLoS One. 2013 Feb 15;8(2):e56603. doi: 10.1371/journal.pone.0056603 (PMC3574070; doi:10.1371/journal.pone.0056603)

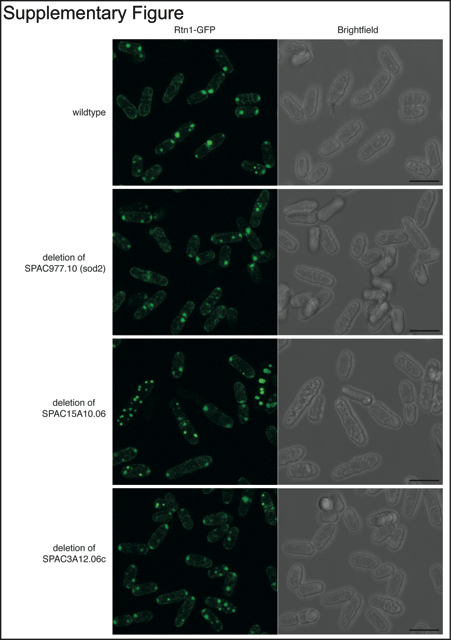

Supplement: Figure S1 — Deletion of sodium channels in fission yeast did not inhibit apogossypol-mediated ER membrane reorganization. Fission yeast strain KT4007 (h90 ade6.M216 leu1 rtn1-GFP-2×FLAG::KanR) carrying GFP-2×FLAG tagged rtn1 gene and subjected to chromosomal gene deletion of either SPAC977.10 (sod2), SPAC15A10.06 or SPAC3A12.06c, still exhibited extensive ER membrane reorganization with apogossypol. (scale bar, 10 µm). Some sort of change in cell morphology (overall larger cells) was noticeable upon deletion of SPAC15A10.06. (TIFF) [file pone.0056603.s001.tiff]
